# Rice straw sample preparation protocol for cell wall major constituent analysis via Raman spectroscopy

**DOI:** 10.1016/j.xpro.2026.104857

**Published:** 2026-09-26

**Authors:** Mahta Mohamadiaza, Naser Farrokhi, Pär K. Ingvarsson, Asadollah Ahmadikhah

**Affiliations:** 1 Department of Cell & Molecular Biology, Faculty of Life Sciences and Biotechnology, Shahid Beheshti University, Tehran 1983969411, Iran; 2 Department of Plant Biology, Swedish University of Agricultural Sciences, 7080 Uppsala, Sweden

**Keywords:** Genetics, Microscopy, Plant sciences, Material science

## Abstract

Raman spectroscopy (RS) is a rapid, label-free, and non-destructive technique for analyzing molecular and spectral characteristics of biological samples. Here, we present a reproducible protocol for glycerol-based sample preparation and RS analysis of long-term air-dried rice straw. We describe steps for preparing samples, acquiring spectral data, and preprocessing data. Our workflow enables reproducible spectral acquisition and semi-quantitative assessment of relative Raman spectral markers associated with major plant cell wall components using standard laboratory procedures. Spectral preprocessing and analysis can be performed with commercially available software, including MATLAB and OriginPro.

## Highlights

• Instructions for glycerol-based tissue preparation for Raman spectroscopy of dried rice stems
• Conditioning steps for improving Raman spectral quality
• Guidance on performing Continuous Wavelet transform-assisted processing for Raman peak identification
• Procedures for peak-based analysis that enables cell-wall marker characterization

## Abstract (editor highlights)

Publisher’s note: Undertaking any experimental protocol requires adherence to local institutional guidelines for laboratory safety and ethics.

## Graphical abstract

**Figure:**
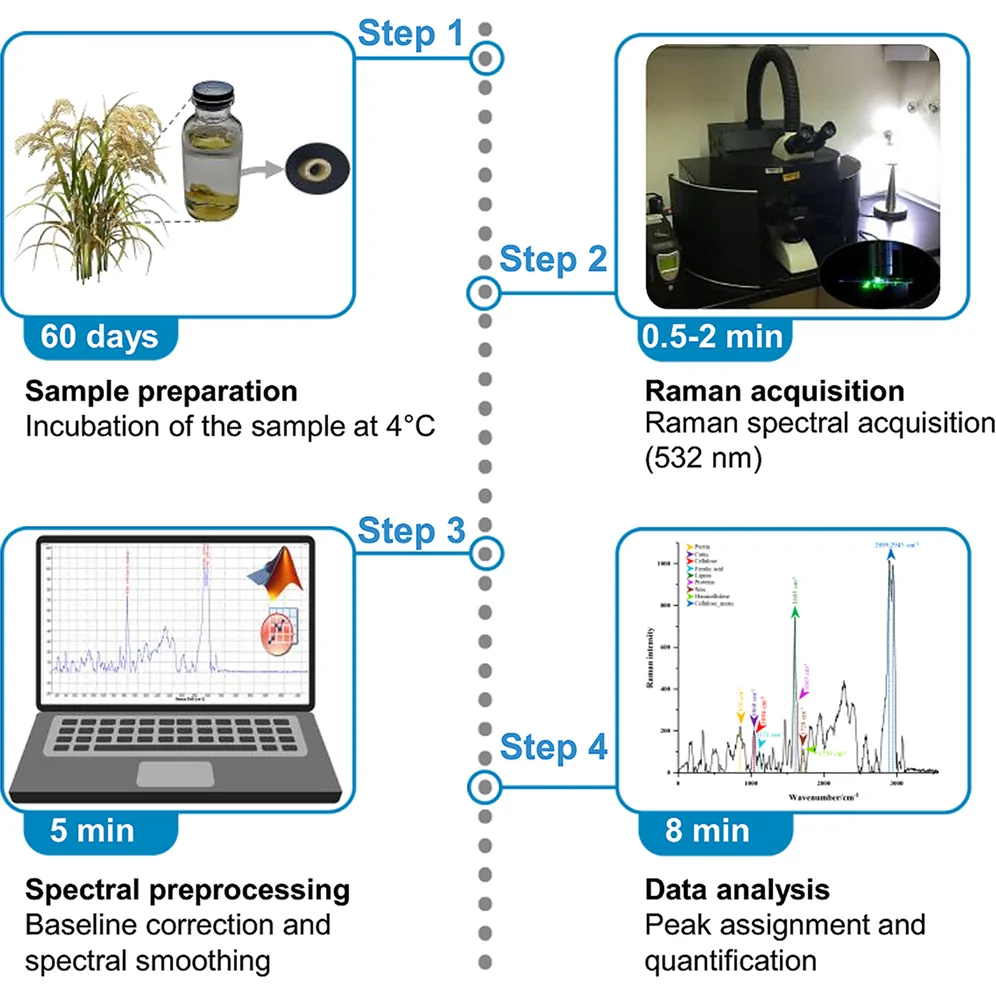

## Before you begin

The plant cell wall is an extensible ultrastructure primarily composed of intertwining polysaccharides, mainly cellulose and matrix polysaccharides, embedded in a pectin-rich matrix, along with structural proteins and enzymes.^1^ Because plant cell walls are structurally complex and chemically heterogeneous, their characterization requires complementary analytical approaches. Cell wall analysis provides information about compositional and structural features, including sugar composition, lignin content, glycosidic bonds, and polymer interactions.^2^ Various techniques used for plant cell wall characterization and their applications and limitations are summarized in Table 1. Among these techniques, RS has been widely applied for the non-destructive investigation of Raman spectral features associated with plant cell walls and other plant tissues.^97^^,^^98^^,^^99^^,^^100^^,^^101^ RS is a photonic technique based on inelastic light scattering.^102^^,^^103^^,^^104^^,^^105^ It is an *in situ*,^106^ rapid, label-free,^107^ and non-destructive method^108^ that provides a spectral fingerprint of a sample, allowing molecular information to be inferred from characteristic Raman features. However, biological samples contain overlapping vibrational signals from multiple polymers, making spectral interpretation challenging.^103^^,^^109^^,^^110^

**Table 1:** Summary of analytical methods commonly used for plant cell wall characterization

Technique	Main applications	Major limitations
UV-Visible spectrophotometry	Major cell wall constituents’ analysis and optical properties	Limited selectivity, Interference from absorbing compounds, Inability to detect non-chromophore molecules^3^^,^^4^^,^^5^^,^^6^^,^^7^^,^^8^^,^^9^^,^^10^^,^^11^^,^^12^^,^^13^
High-performance anion-exchange chromatography with pulsed amperometric detection (HPAEC-PAD)	Carbohydrate composition and monosaccharide analysis	High cost, Complex operation, Long analysis times for electrode equilibration^14^^,^^15^^,^^16^^,^^17^^,^^18^^,^^19^^,^^20^^,^^21^^,^^22^^,^^23^^,^^24^^,^^25^^,^^26^^,^^27^^,^^28^^,^^29^^,^^30^^,^^31^^,^^32^
Gas chromatography–mass spectrometry/flame ionisation detection (GC–MS/FID)	Lignin and carbohydrate composition analysis	Requires derivatization, Time-consuming, Limited quantitative performance for some complex samples^22^^,^^32^^,^^33^^,^^34^^,^^35^^,^^36^^,^^37^^,^^38^^,^^39^^,^^40^^,^^41^^,^^42^
Liquid chromatography electrospray ionisation tandem mass spectrometry (LC–ESI–MS/MS)	Structural characterization of oligosaccharides and other cell wall biomolecules	High cost, ion suppression Limited sample throughput^30^^,^^43^^,^^44^^,^^45^^,^^46^^,^^47^^,^^48^^,^^49^^,^^50^^,^^51^
One- (1D), two- (2D) and three (3D) dimensional nuclear magnetic resonance (NMR)	Structural characterization of cell wall polymers	Time-consuming, Limited sensitivity, Expensive instrumentation^52^^,^^53^^,^^54^^,^^55^^,^^56^^,^^57^^,^^58^^,^^59^^,^^60^^,^^61^^,^^62^^,^^63^^,^^64^^,^^65^^,^^66^
Infra-red (IR) spectroscopy and comprehensive microarray polymer profiling (CoMPP)	Analysis of cell wall composition, functional groups, and polysaccharide distribution	IR: spectral interpretation can be challenging, particularly for hydrated samples; CoMPP: High cost, Limited probe coverage^67^^,^^68^^,^^69^^,^^70^^,^^71^^,^^72^
Four-dimensional fluorescence confocal imaging	Three-dimensional visualization of cell wall architecture and dynamics	Limited imaging depth, Phototoxicity and Complex sample preparation^73^^,^^74^^,^^75^^,^^76^^,^^77^^,^^78^
Light microscopy	Visualisation of cell morphology and tissue structure	Limited spatial resolution Staining may be required^79^^,^^80^^,^^81^^,^^82^^,^^83^
Scanning electron microscopy (SEM)	Cell wall surface morphology	Requires specialized sample preparation and instrumentation; Unsuitable for live samples^84^^,^^85^^,^^86^^,^^87^^,^^88^^,^^89^^,^^90^
RS	Label-free chemical and structural characterization of plant cell wall components	Weak Raman signal, fluorescence interference Limited depth resolution, Potential laser-induced sample alteration^91^^,^^92^^,^^93^^,^^94^^,^^95^^,^^96^

Raman microscopy has been applied to plant tissues to investigate Raman spectral features associated with cell wall components in their native state, with spatial resolution at the cellular and cell wall levels.^111^ This technique has been used to study the distribution of cellular components, including nucleic acids, proteins, lipids, and carbohydrates.^112^^,^^113^ Because multiple polymers contribute simultaneously to plant cell wall spectra, appropriate spectral preprocessing and peak-based interpretation are required.

This protocol describes a workflow for preparing long-term dried rice stem (straw) samples for Raman micro-spectroscopy and subsequent peak-based semi-quantitative analysis of Raman spectral markers associated with major plant cell wall components. The protocol is intended for characterisation at the tissue level rather than for the analysis of individual cell types or specific cell wall layers. It is specifically optimized for plant tissues that have been air-dried for extended periods (≥1 year), which are typically difficult to analyze due to brittleness and low moisture content. Unlike conventional cryosection-based approaches, the proposed protocol does not require specialized frozen-sectioning equipment, providing a simple and practical alternative for preparing plant tissues for Raman analysis.

Although this protocol was developed and validated using rice stem tissues, it may also apply to other cereal crops and other grasses with similar structural characteristics. However, this has not yet been experimentally validated.

### Innovation

Recent protocols have focused on specific aspects of rice straw processing, such as lignocellulose visualisation following alkaline pretreatment for methane production. However, a standardized sample preparation protocol for Raman microspectroscopic analysis of long-term dried rice straw has remained unavailable.

The principal innovation of this protocol is the introduction of a glycerol–water conditioning step for long-term dried rice straw before Raman microspectroscopy. This pretreatment restores the flexibility of brittle, low-moisture tissues, facilitates sample handling, and enables the acquisition of high-quality and reproducible Raman spectra from samples that are otherwise difficult to analyze.

The Raman spectral acquisition, preprocessing, peak assignment, and semi-quantitative analysis based on diagnostic Raman bands employed in this protocol are based on established methodologies.

The methodological advance presented here lies in adapting these well-established Raman procedures to long-term dried rice straw through an optimized sample preparation workflow.

Combining this sample preparation strategy with a standardized workflow for Raman measurements and data analysis, the protocol enables semi-quantitative assessment of Raman spectral markers associated with major lignocellulosic components in diverse rice genotypes, including samples stored for extended periods (*e.g*., herbarium specimens). This expands the applicability of RS to archived plant materials and facilitates comparative studies based on Raman spectral characteristics of biomass samples.

### Sample preparation setup

**Timing: 1 h**

This step conditions long-term dried rice stem tissues using 50% (v/v) solution of distilled water and glycerol.

1. Collect rice stem (straw) samples from the third internode (counted from the top) of rice plants that have been air-dried for a prolonged period (≥1 year).
2. Prepare a 1:1 (v/v) solution of distilled water and glycerol.
3. Label appropriate glass containers for sample incubation.
4. Set a refrigerator or cold room to 4°C for sample storage during incubation.

### Pre-incubation considerations

**Timing: 5 min**

This step prepares samples for incubation.

5. Cut dried stem samples into approximately 2 cm segments before immersion.

**CRITICAL:** Ensure that the prepared glycerol solution volume is sufficient to fully immerse all samples.

***Note:*** The volume of solution should be approximately ten times the sample volume to ensure complete coverage.

6. Place samples in labeled glass containers containing the glycerol solution and ensure complete immersion.

**CRITICAL:** Incomplete immersion may lead to uneven rehydration and reduced spectral quality.

### Handling and storage considerations

**Timing: 30 s**

This step ensures proper handling of samples during incubation.

7. Sample incubation.
  a. Monitor samples periodically during incubation.
    **CRITICAL:** If any visible color change occurs in the solution, transfer samples to fresh solution with the same composition.
  b. For large sample sets, divide samples into multiple groups and prepare them on consecutive days.
    ***Note:*** This approach helps maintain consistency during subsequent Raman spectral acquisition.

## Key resources table

**Table:** 

REAGENT or RESOURCE	SOURCE	IDENTIFIER
**Biological samples**		

Rice stem samples (*Oryza sativa* L.)	International Rice Research Institute (IRRI)	N/A

**Chemicals, peptides, and recombinant proteins**		

Glycerol	Laboratory stock	N/A
Distilled water	Laboratory supply	N/A

**Software and algorithms**		

MATLAB	MathWorks	R2020a
OriginPro	OriginLab	OriginPro 2021
MATLAB code for CWT-based peak enhancement and Raman spectral preprocessing	This work	Data_S1.pdf

**Other**		

Raman microspectrometer	Teksam	Takram P50C0R10
Optical microscope	Teksam system integrated	N/A

## Materials and equipment

Raman spectral data are acquired from 147 rice genotypes. One conditioned stem segment (collected from the third internode) is analyzed for each genotype, and one Raman spectrum is acquired from each sample. Each spectrum is considered a single spectral observation, representing the corresponding genotype. After quality assessment following baseline correction, 17 spectra exhibiting excessive residual noise are excluded. Consequently, 130 genotypes are retained for downstream statistical analysis.

Genotype information for 130 rice samples used in this study, including subpopulation classification and sample identifiers, is provided in Table S1 (supplemental information).

## Step-by-step method details

### Sample conditioning and glycerol-based tissue preparation for RS

**Timing: 60 days**

This step conditions long-term dried rice stem tissues using 50% (v/v) aqueous glycerol immersion to restore tissue flexibility and enable reproducible high-quality Raman spectral acquisition.

***Note:*** The selection of 50% (v/v) glycerol was based on our previous work, in which the same conditioning solution successfully improved the preparation of long-term dried rice tissues for scanning electron microscopy.^114^

Preliminary evaluation of alternative conditioning solutions demonstrated that 50% (v/v) glycerol produced the most suitable Raman spectra for subsequent optimization of incubation time (Figure S1).

The 60-days incubation period was selected based on empirical optimization. Raman spectra acquired after different incubation periods (7, 14, 20, 40, 60, 80, and 110 days; Figure S1) showed progressive improvement up to approximately 60 days, with no substantial additional changes thereafter. Therefore, 60 days was selected as the shortest incubation period that consistently produced stable Raman spectra.

1. Cut the collected stem samples into approximately 2 cm segments.
2. Prepare a 1:1 (v/v) water: glycerol solution.
3. Place the stem segments in labeled glass containers.
4. Add sufficient solution to completely immerse all samples.
5. Incubate samples at 4 °C for 60 days.

**CRITICAL:** Because the samples are fully dried, ensure complete immersion during incubation. This step is essential for tissue conditioning and maintaining sample integrity during the prolonged preparation period, and it improves Raman spectral acquisition. Incubation at 4 °C was used to minimize the risk of microbial growth.

***Note:*** Samples with sufficient phenotypic diversity (Figure 1; Table S2) have been successfully prepared and analyzed using RS with this approach; however, the applicability of this protocol to different rice stem tissues or other plant materials requires further experimental validation.

***Note:*** The solution volume must completely cover each sample to ensure full immersion. To satisfy this requirement, use a solution volume approximately ten times the sample volume (Figure 2).

***Note:*** To prevent excessive dryness and brittleness and to obtain smoother sectioning surfaces, prepare smaller sections two days after placing samples in the preparation solution.

**CRITICAL:** Monitor samples during incubation. If any visible color change is observed in the solution, transfer samples to a fresh container with the same solution composition.

**Pause point:** Conditioned samples can be maintained in 50% (v/v) glycerol solution at 4 °C after the 60-days incubation period until Raman spectral acquisition.

**Figure 1:**
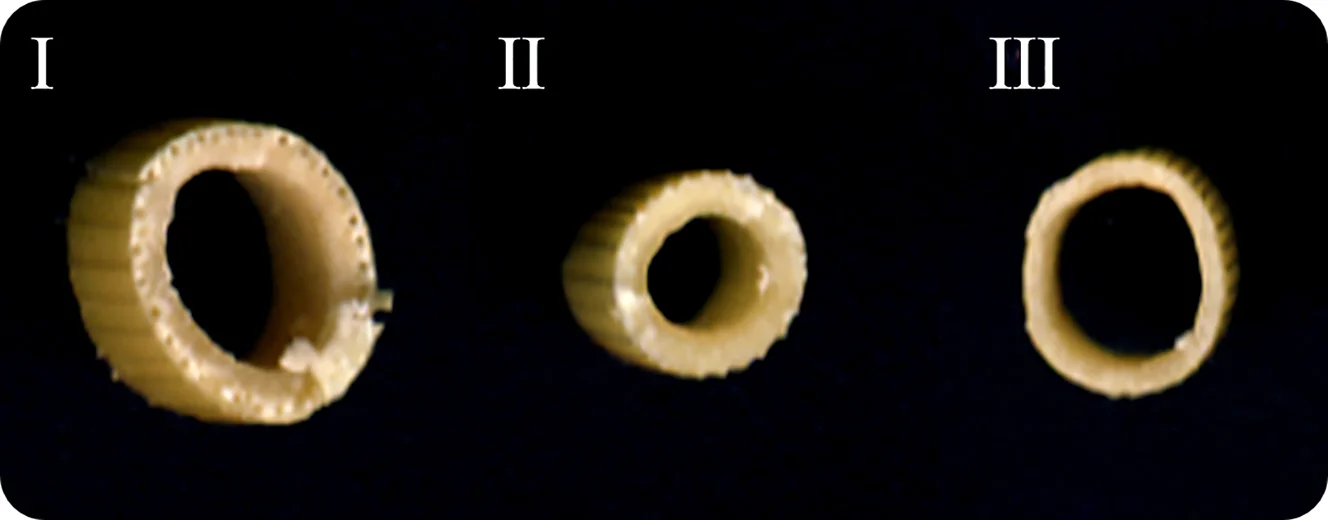
Representative rice straw samples with distinct phenotypic characteristics Sample I (g236), Sample II (g57), and Sample III (g135) are shown (see Table S2).

**Figure 2:**
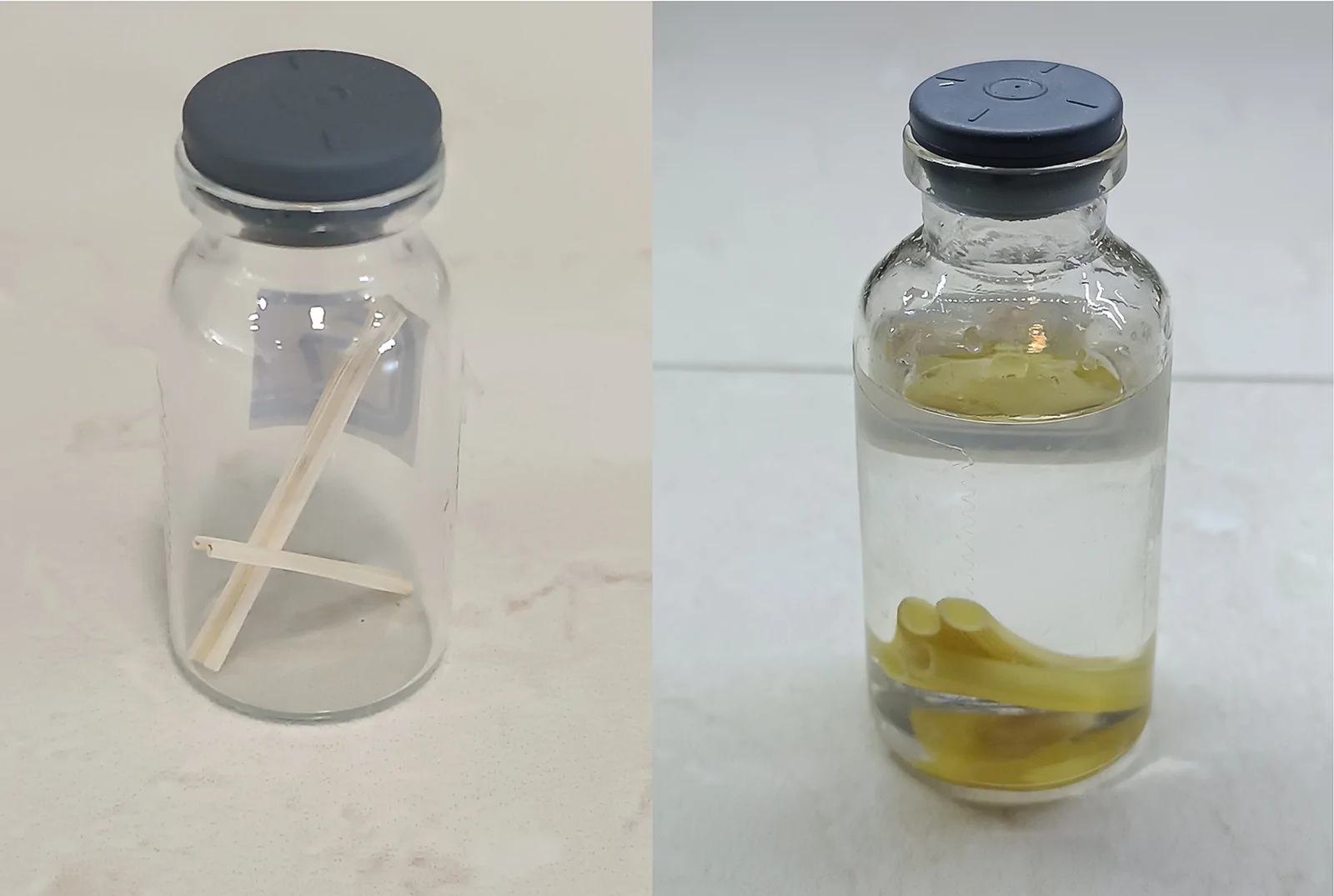
Complete immersion of rice straw samples in 50% (v/v) glycerol solution during incubation

Use the prepared samples for Raman spectral acquisition and data pre-processing in the following step.

### Spectral acquisition and data pre-processing

**Timing: 7 min**

This step acquires spectral data and performs data preprocessing.

Prepared samples from the previous step are analyzed using RS.

Micro-RS combines an optical microscope with a Raman spectrometer, enabling high spatial resolution (∼1 μm) sampling.^115^ It provides a non-destructive and location-specific approach for investigating molecular and structural characteristics of cellular and tissue components through their Raman vibrational signatures.^116^

In this study, a Raman microspectrometer (Takram P50C0R10, Teksam; Figure 3A) equipped with a 532 nm Nd:YAG laser was used in point-to-point scanning mode over a spectral range of −52 to 4600 cm^−1^. The laser is focused onto a small spot on the sample, and the Raman signal is collected from that location to generate a Raman spectrum.

**CRITICAL:** Ensure that the samples equilibrate to laboratory temperature (approximately 20–25°C) before spectrometry to minimize temperature-related variation during spectral acquisition.

**CRITICAL:** When processing many samples, gradually transfer them from 4 °C to ambient temperature to avoid thermal stress.

6. Spectral acquisition. Follow the steps below according to.^117^
  a. Turn on the Raman microspectrometer and open the instrument operating software.
  b. Calibrate and align the spectrometer using an appropriate calibration source.
  c. Mount the sample on the microscope stage for spectral acquisition.
    ***Note:*** Conditioned stem segments (approximately 2 cm in length) collected from the third internode^118^ were placed horizontally on a standard glass microscope slide. Spectra were acquired from the longitudinal surface and represent the analyzed tissue region rather than a specific cell type.
  d. Use the microscope at the selected magnification to examine the sample and focus on the region of interest before spectral acquisition.
  e. Set Raman acquisition parameters, including excitation wavelength, laser power, acquisition time, and spectral range, according to the conditions specified in this protocol.
    ***Note:*** Use a laser power meter to determine the laser power delivered to the sample.
  f. Acquire Raman spectra using an exposure time optimized for each sample (0.5–2 min per spectrum in this study).
  g. Save all measurements before data processing and convert them to a universal format if required (Figure 3B).
    ***Note:*** Data sets can be stored before data processing.
    ***Note:*** The wavelength of the laser is critical to the experimental design. Raman maps can be obtained by raster-scanning of the laser line focused across the sample. Line mapping can be achieved in shorter acquisition times compared with point mapping, without compromising image quality.^119^^,^^120^^,^^121^ However, the intensity varies along the laser line, and therefore, this approach results in spectra with varying SNR along the laser line. As an alternative method, global illumination of the sample area by a high-brightness lamp results in high-quality spectral imaging with improved power distribution across the sample (Figure 3A).
    ***Note:*** Some samples may require longer laser exposure times due to small volume or weak scattering properties.^117^ In this study, depending on structural characteristics, laser exposure time (λ = 532 nm) was set between 0.5 and 2 min.
7. Pre-processing. Immediately after acquiring the spectra, assess spectral quality and apply preprocessing steps to minimize non-biological variability and improve downstream analysis.^122^
  a. Optimize Raman signal quality by adjusting spectrometer settings, including increasing integration time and applying optical bleaching to the sample.
  b. Perform baseline correction using OriginPro software to remove fluorescence background and slowly varying baseline contributions from Raman spectra. Apply the same baseline correction method and fitting parameters for all spectra to ensure reproducibility.
    ***Note:*** Baseline correction is a widely used step in Raman spectral preprocessing. It refers to either the removal of substrate-derived Raman signals or fluorescence baseline correction. The former removes substrate contributions when they exhibit significant Raman features, especially when overlapping with sample peaks. The latter removes fluorescence backgrounds that appear as slowly varying baselines beneath Raman bands.^103^^,^^123^^,^^124^
    ***Note:*** Because Raman spectra of plant cell wall components often contain multiple overlapping bands, continuous wavelet transform (CWT)-based peak enhancement can be applied before peak extraction to improve peak identification and reduce the risk of incorrect peak assignment during semi-quantitative analysis.
  c. Using OriginPro software, truncate spectra to the defined spectral range (0–3500 cm^−1^) to focus on relevant Raman peaks for rice stem samples (Figure 3C).
    **CRITICAL:** When processing a large number of samples that cannot be analyzed in a single day, divide samples into multiple groups and prepare them on consecutive days. Apply the same grouping strategy during RS acquisition.
    ***Alternatives:*** Spectral acquisition can be postponed beyond 60 days if necessary. In such cases, incubation can be extended for at least 20 additional days, provided that the solution is refreshed after day 60 (Figures 4 and 5).
  d. Apply CWT-based peak enhancement to the spectral data using the provided MATLAB code before diagnostic Raman band extraction and peak-based semi-quantitative analysis (Figure 6).
    ***Note:*** The CWT coefficient signal enhances diagnostically relevant spectral features and improves the discrimination of closely spaced Raman bands. In this study, the CWT is used as a feature-enhancement technique for peak detection rather than as a wavelet denoising or spectral smoothing method. The MATLAB implementation follows the CWT-based peak enhancement framework described by Liu et al.^125^ (supplemental information).
    %% CWT-Based Raman Peak Enhancement and Peak Detection
    % Purpose -------
    % This script demonstrates the use of the Continuous Wavelet Transform (CWT) as a feature-enhancement technique for Raman peak detection.
    % IMPORTANT ---------
    % This implementation DOES NOT perform wavelet denoising or signal reconstruction (icwt). The CWT coefficient signal is used only to
    % enhance peak-related spectral features before automatic peak detection.
    % Input -----
    % Excel file with two columns:
    % Column 1 : Raman Shift (cm^∧^-1)
    % Column 2 : Raman Intensity
    % Output ------
    % 1. Publication-quality figure
    % 2. Table of detected Raman peaks
    % 3. High-resolution PNG image
    % ========================================================================
    clc
    clear
    close all
    %% ========================================================================
    % USER-ADJUSTABLE PARAMETERS
    % ========================================================================
    InputFile = 'MatlabInput.xlsx';
    % Mother wavelet: 'mexh' = Mexican Hat wavelet; Suitable for narrow Raman peaks.
    WaveletName = 'mexh';
    % Range of wavelet scales
    Scales = 1:30;
    % Selected scale used for peak enhancement
    TargetScale = 5;
    % Relative threshold
    ThresholdFactor = 0.20;
    % Peak detection parameters
    MinPeakProminence = 0.05;
    MinPeakDistance = 5;
    % Plot range
    PlotRange = [0 3500];
    % Export figure?
    SaveFigure = true;
    FigureName = 'Figure6_CWT_PeakEnhancement.png';
    %% ========================================================================
    % LOAD RAMAN SPECTRUM
    % ========================================================================
    fprintf('\n');
    fprintf('Reading Raman spectrum.\n');
    Data = readmatrix(InputFile);
    Wavenumber = Data(:,1);
    Intensity = Data(:,2);
    fprintf('Number of spectral points : %d\n',length(Intensity));
    %% ========================================================================
    % CONTINUOUS WAVELET TRANSFORM
    % ========================================================================
    fprintf('\n');
    fprintf('Computing Continuous Wavelet Transform.\n');
    CWTcoeff = cwt(Intensity,Scales,WaveletName);
    %% ========================================================================
    % SELECT CWT COEFFICIENT SIGNAL
    % ========================================================================
    % The selected scale is used only for feature enhancement. This is NOT a reconstructed Raman spectrum.
    CoefficientSignal = real(CWTcoeff(TargetScale,:));
    %% ========================================================================
    % THRESHOLD LOW-AMPLITUDE COEFFICIENTS
    % ========================================================================
    CoefficientThreshold = ThresholdFactor∗max(abs(CoefficientSignal));
    oefficientSignal(abs(CoefficientSignal)<CoefficientThreshold)=0;
    %% ========================================================================
    % PEAK DETECTION
    % ========================================================================
    fprintf('Detecting Raman peaks.\n');
    [PeakHeight,PeakPosition] = findpeaks(CoefficientSignal,Wavenumber,'MinPeakProminence',MinPeakProminence,'MinPeakDistance',MinPeakDistance);
    fprintf('Detected Peaks = %d\n',length(PeakPosition));
    %% ========================================================================
    % PLOT RESULTS
    % ========================================================================
    figure('Color','w','Position',[200 80 850 900]);
    %% ------------------------------------------------------------------------
    subplot(3,1,1)
    plot(Wavenumber,Intensity,'k','LineWidth',1.2)
    xlabel('Raman Shift (cm^∧^{-1})')
    ylabel('Intensity (a.u.)')
    title('Original Raman Spectrum')
    xlim(PlotRange)
    grid on
    box on
    set(gca,'FontName','Times New Roman','FontSize',11)
    %% ------------------------------------------------------------------------
    subplot(3,1,2)
    plot(Wavenumber,CoefficientSignal,'r','LineWidth',1.3)
    hold on
    plot(PeakPosition,PeakHeight,'ko','MarkerFaceColor','y','MarkerSize',7)
    xlabel('Raman Shift (cm^∧^{-1})')
    ylabel('CWT Coefficient')
    title(['CWT-Based Peak Enhancement (Scale = ',num2str(TargetScale),')'])
    xlim(PlotRange)
    grid on
    box on
    set(gca,'FontName','Times New Roman','FontSize',11)
    %% ------------------------------------------------------------------------
    subplot(3,1,3)
    imagesc(Wavenumber,Scales,abs(CWTcoeff));
    axis xy
    xlabel('Raman Shift (cm^∧^{-1})')
    ylabel('Wavelet Scale')
    title('Continuous Wavelet Transform Coefficient Map')
    colormap(jet)
    colorbar
    set(gca,'FontName','Times New Roman','FontSize',11)
    %% ========================================================================
    % SAVE FIGURE
    % ========================================================================
    if SaveFigure
    print(gcf,FigureName,'-dpng','-r1200');
    end
    ***Note:*** The MATLAB script provided with this protocol is intended to demonstrate the proposed spectral preprocessing and CWT-based peak enhancement workflow using a representative Raman spectrum. The script includes the complete sequence of data import, CWT-based peak enhancement, peak detection, and visualization of the processing results. Baseline correction and spectral truncation are performed separately using OriginPro software as described above. All user-adjustable parameters, including the wavelet type, wavelet scale, threshold factor, and peak-detection settings, are explicitly defined within the script and may be modified according to the characteristics of different Raman datasets (supplemental information).

**Figure 6:**
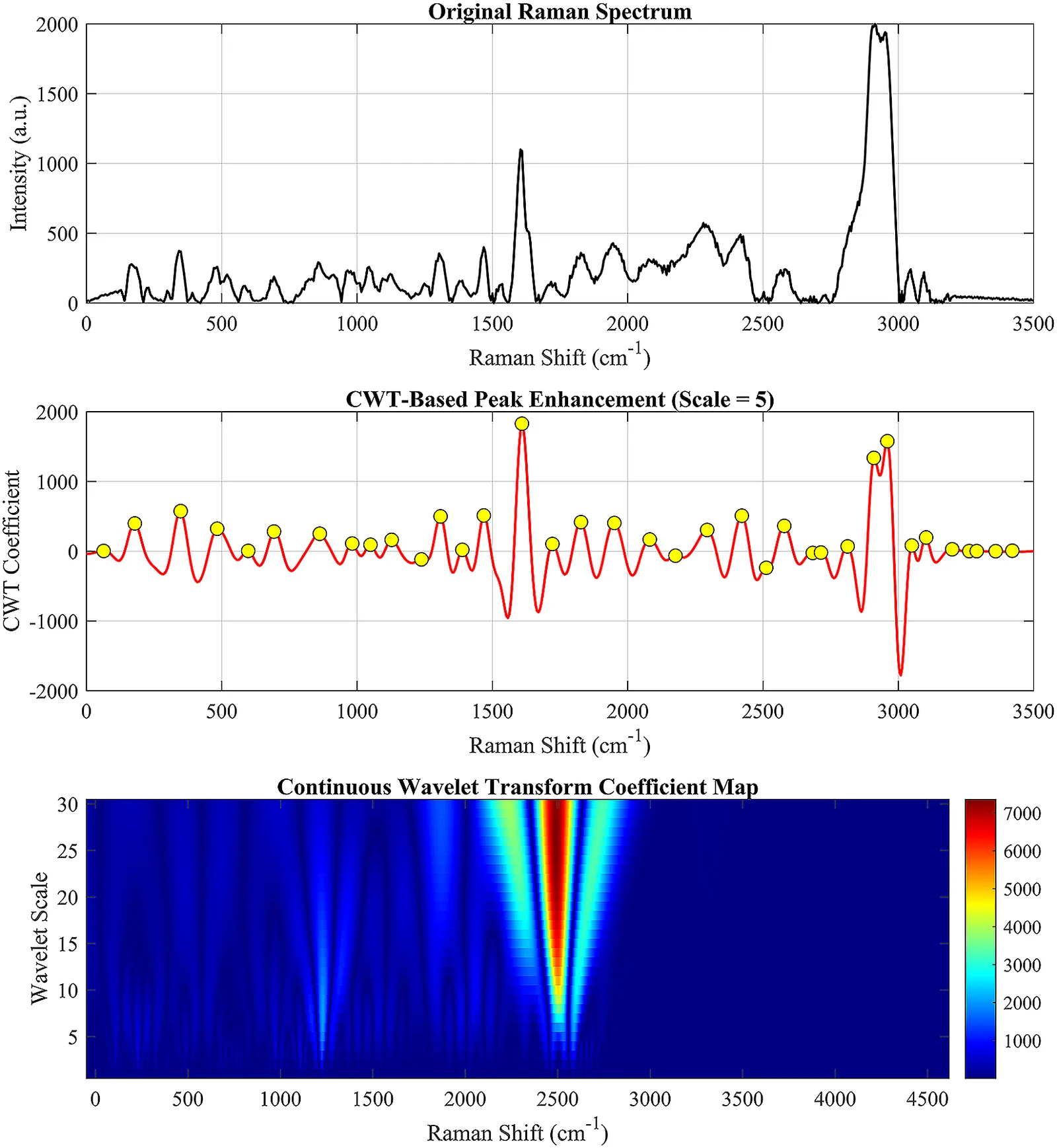
CWT-based peak enhancement following the methodology of Liu et al.^125^ (top) Baseline-corrected Raman spectrum of sample g135. (middle) CWT coefficient signal at the selected wavelet scale used for peak enhancement and subsequent peak detection. This panel represents the CWT coefficient response rather than a reconstructed or denoised Raman spectrum. (bottom) CWT coefficient map of the Raman spectrum shown in (A), illustrating the multiscale representation of spectral features.

**Figure 4:**
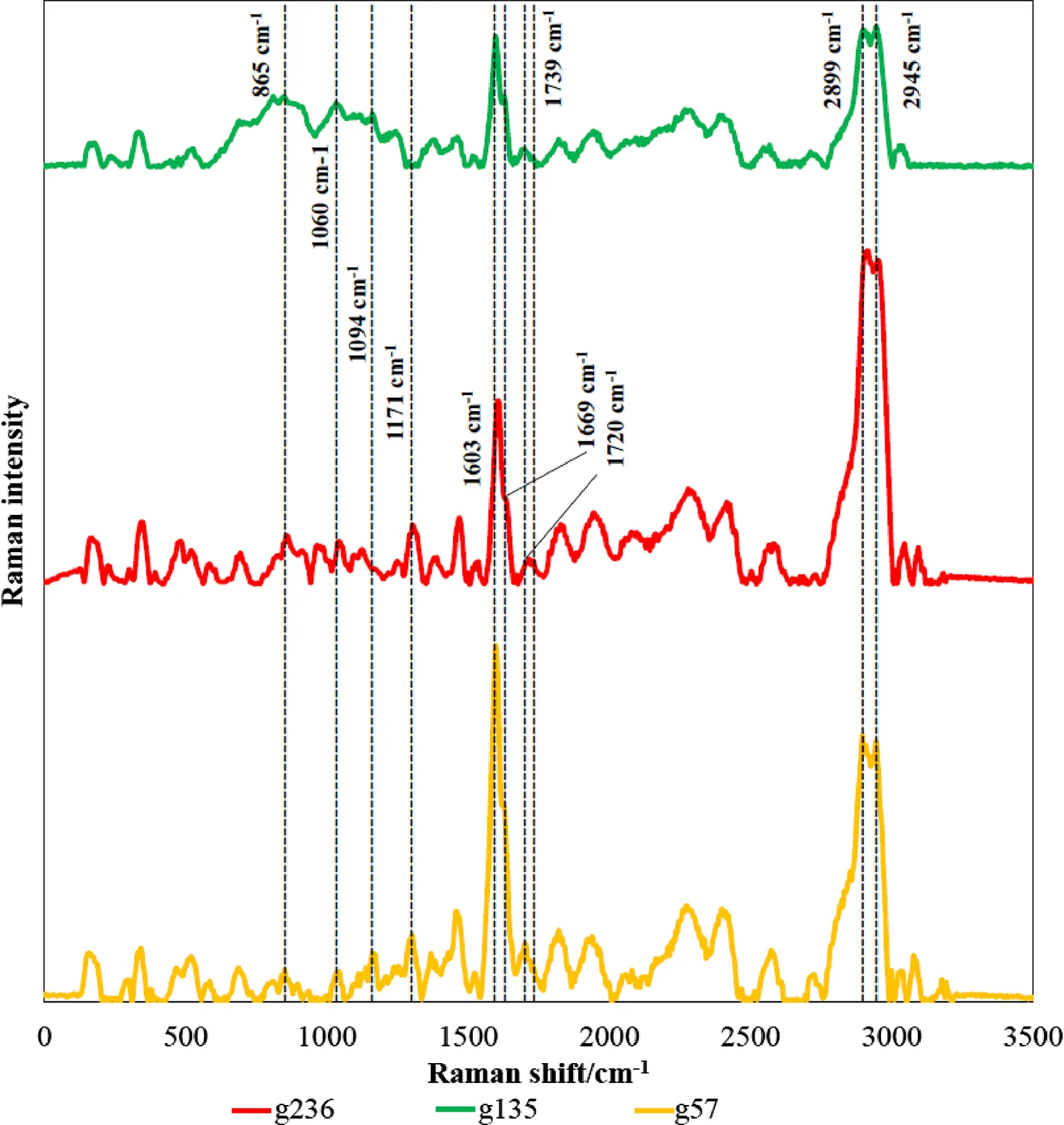
Effect of incubation in 50% (v/v) glycerol at 4 °C on Raman spectral quality after 60 days Optimal spectral quality, characterized by well-resolved and high-intensity peaks, is observed under these conditions.

**Figure 5:**
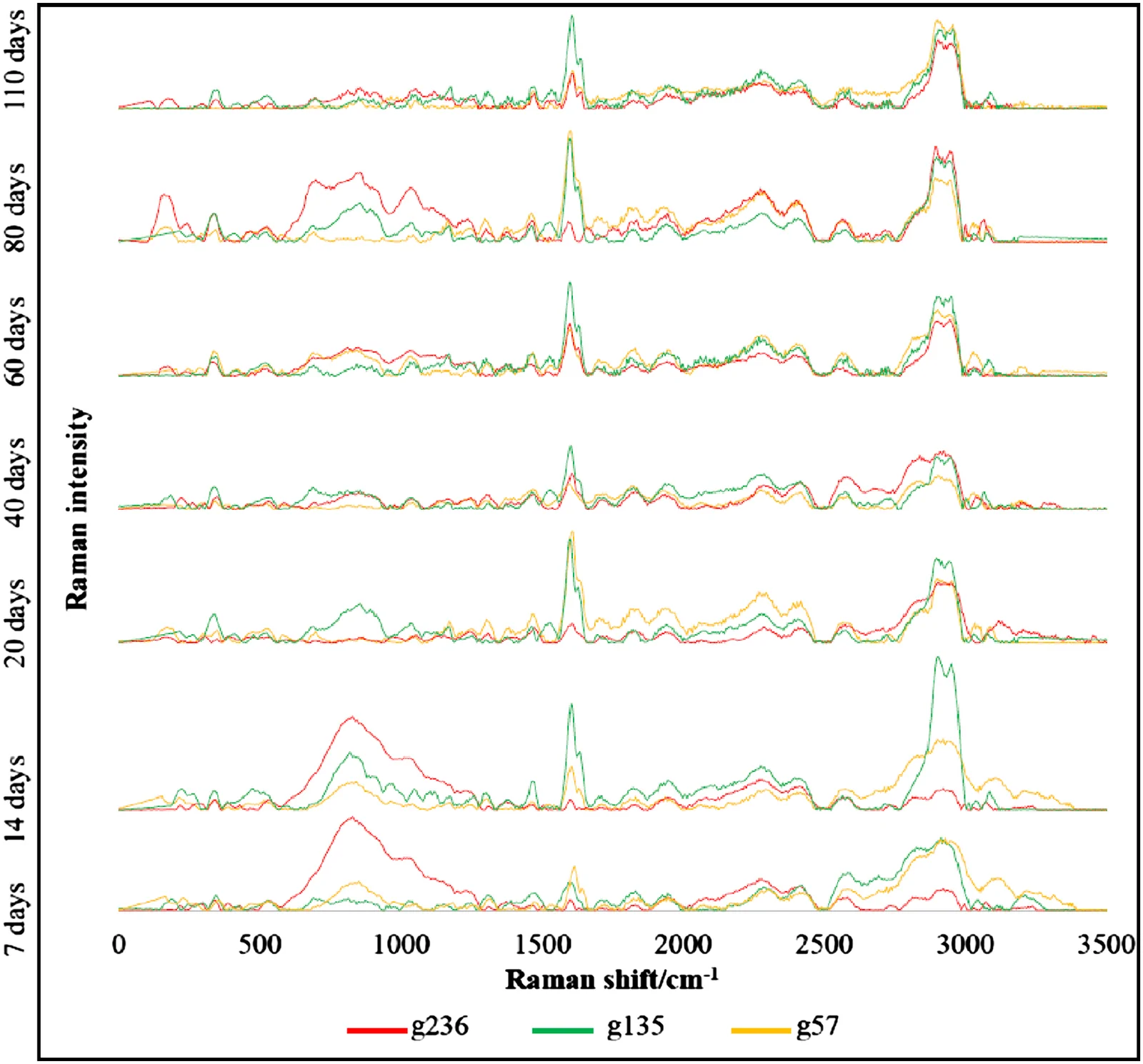
Representative Raman spectra of rice stem samples acquired after different incubation periods in 50% (v/v) glycerol (7, 14, 20, 40, 60, 80, and 110 days) The spectra demonstrate a progressive improvement in spectral quality with increasing incubation time, with no substantial additional changes observed after 60 days, supporting the selection of 60 days as the optimal conditioning period.

**Figure 3:**
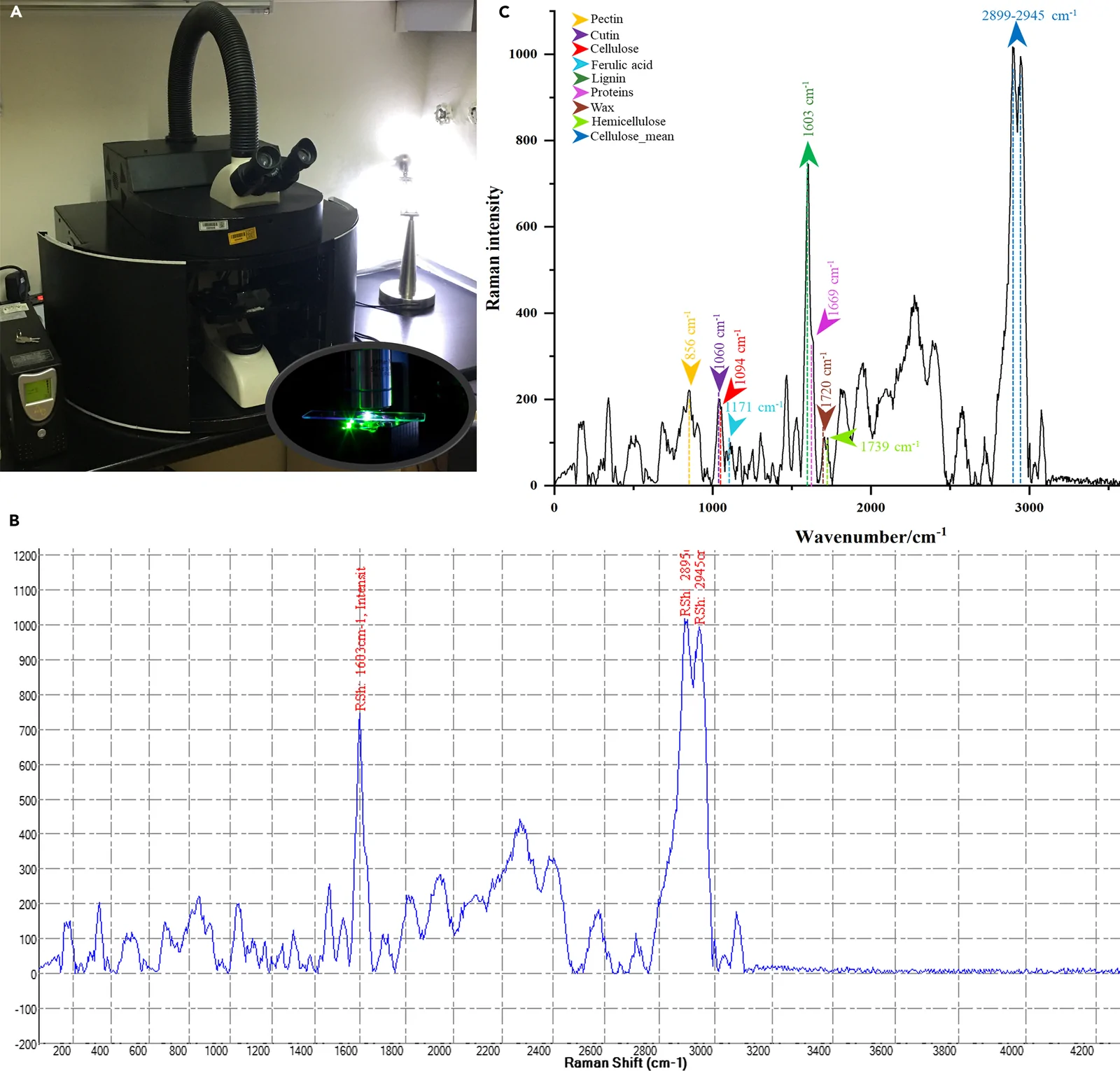
Peak-based semi-quantitative analysis of Raman spectral markers associated with rice straw cell walls Different spectra are shown in distinct colors representing Raman spectral markers associated with eight major components: pectin, cutin, cellulose, ferulic acid, lignin, proteins, waxes, and hemicellulose. Spectral acquisition parameters include excitation wavelength (λ = 532 nm), laser power output (70 mW), and baseline correction. (A) Raman microspectrometer setup (Takram P50C0R10). (B) Representative raw Raman spectrum obtained by micro-Raman spectroscopy. (C) Processed Raman spectrum showing the diagnostic Raman bands used for the peak-based semi-quantitative analysis of Raman spectral markers associated with major plant cell wall components after preprocessing and visualization in OriginPro software.

### Spectral analysis and semi-quantitative analysis

**Timing: 8 min**

This step describes data analysis.

8. Use the information provided in Table 2 to perform semi-quantitative analysis of Raman spectra. Evaluate relative Raman spectral markers associated with eight major plant cell wall constituents based on the position, intensity, and shape of diagnostic Raman bands, (Figures 3C; Table S3).

**Table 2:** Assignment of Raman spectral bands associated with plant cell wall components based on literature sources

Traits	Band (cm^−1^)	In this study	Assignment
Pectin (Pec)	854–856	856	C–O–C skeletal mode of α-anomers of low methylated pectin^126^^,^^127^^,^^128^^,^^129^^,^^130^^,^^131^^,^^132^^,^^133^
Cutin (Cut)	1061	1060	C-C stretching^127^^,^^134^
Cellulose (Cell_1094)	1093–1097	1094	asymmetric stretching of glycosidic C–O–C^127^^,^^130^^,^^131^^,^^132^^,^^133^^,^^135^
Ferulic acid (Fer)	1171	1171	C-C and C–O stretching^127^^,^^136^^,^^137^
Lignin (Lig_max)	1600–1605	1597–1603	aromatic ring stretch^127^^,^^130^^,^^138^
Proteins (Pro)	1670	1669	amide bonds (R-C(=O)-N-R)^127^^,^^139^
Cuticular wax (Wax)	1720	1720	(C¼O stretching)^127^^,^^140^
Hemicellulose (Hemi)	1739	1739	C=O stretching vibration^126^^,^^129^^,^^130^^,^^141^
Cellulose (Cell_mean)	2897–2943	2899–2945	CH and CH2 stretching^127^^,^^130^^,^^131^^,^^132^^,^^133^^,^^135^

***Note:*** Peak assignment was based on diagnostic Raman bands consistently reported for each plant cell wall component in multiple literature sources. Spectral regions with potential overlap among different cell wall constituents or with glycerol-related signals were interpreted with caution and were not used as the sole basis for Raman spectral marker assignment.

***Note:*** In addition to peak position, peak shape contains significant information that can reveal structural characteristics and spectral variations of the material. Cell wall component analysis relies heavily on spectral libraries containing reference spectra.^127^

***Note:*** Because the conditioning solution contains 50% (v/v) glycerol, interpret spectral regions with potential glycerol overlap cautiously. Raman spectral marker assignment should rely primarily on well-established diagnostic Raman bands consistently reported across multiple literature sources.

***Note:*** In this protocol, spectral analysis does not require extensive sample preparation. Raman spectral features related to biochemical and structural characteristics can be obtained without labeling and by relying on reference-based spectral comparison.

***Note:*** Multiple Raman spectral references are available, as described in Table 2. The reported values represent relative Raman peak intensities derived from diagnostic bands rather than absolute chemical concentrations.

## Expected outcomes

This protocol yields high-quality Raman spectra from long-term dried rice stem tissues, including samples stored for extended periods that are typically difficult to analyze due to brittleness and low moisture content. The resulting spectra exhibit sharp, well-resolved peaks with improved signal-to-noise ratios following sample conditioning and spectral preprocessing.

The acquired spectra enable the evaluation of Raman spectral markers associated with major plant cell wall components, including cellulose, hemicellulose, lignin, pectin, cutin, waxes, and proteins, based on the position, intensity, and shape of diagnostic Raman bands. The processed data are suitable for qualitative interpretation and peak-based semi-quantitative evaluation of relative Raman spectral variations associated with these biochemical components.

The resulting datasets are compatible with downstream multivariate and genetic analyses, such as genome-wide association studies (GWAS; unpublished data). In addition, the protocol produces reproducible spectral datasets across samples with diverse morphological and genotypic characteristics, supporting comparative analyses based on Raman spectral characteristics across plant materials (Table S3).

Overall, this approach enables the extraction of Raman-based biochemical and structural information from plant tissues and facilitates reference-based spectral comparison for Raman marker interpretation and semi-quantitative analysis.

## Quantification and statistical analysis

Raman spectral data are pre-processed as described in the *Spectral acquisition and data pre-processing* section, including baseline correction, optional CWT-based peak enhancement for peak identification, and spectral range truncation (0–3500 cm^−1^). CWT is used to enhance peak-related spectral features and improve discrimination of closely spaced Raman bands prior to peak detection rather than to generate a denoised or smoothed spectrum. The processed spectra are subsequently used for peak-based semi-quantitative and statistical analyses.

For peak intensity extraction, Raman spectra are subjected to baseline correction to remove background fluorescence. The intensity values of selected diagnostic Raman bands are extracted from baseline-corrected spectra using peak maxima (peak height) at predefined wavenumbers listed in Table 2 (Table S3). CWT-based peak enhancement is applied as an optional preprocessing step to facilitate peak identification by enhancing peak-related spectral features before automatic peak detection. This implementation follows the CWT-based peak enhancement strategy proposed by Liu et al,^125^ in which CWT coefficients are used for peak localization rather than reconstruction of a denoised spectrum. Peak intensity values used for semi-quantitative analysis is extracted directly from baseline-corrected spectra rather than from CWT coefficient signals. No peak fitting, peak area integration, spectral deconvolution, or inverse wavelet reconstruction is applied for peak intensity-based semi-quantitative analysis.

The selection of an appropriate preprocessing technique depends on the characteristics of the measured spectra and the objective of the subsequent analysis. Because Raman spectra contain closely spaced and partially overlapping bands, CWT-based peak enhancement was selected to improve peak localization while preserving spectral characteristics. CWT is used only for feature enhancement and peak detection and is not applied for spectral denoising, smoothing, or reconstruction.

Semi-quantitative analysis is performed based on the position, intensity, and shape of predefined diagnostic Raman bands corresponding to Raman spectral markers associated with eight major cell wall components (Table 2; Table S3). Peak intensities are extracted from baseline-corrected spectra and used as variables for subsequent statistical analyses.

Statistical analyses are conducted to assess variability among samples and genotypes. Multivariate statistical approaches are applied to explore relationships between Raman spectral markers and cell wall-related spectral variation. Genome-wide association studies (GWAS) are performed using derived spectral indices as phenotypic traits (unpublished data; associated manuscript in preparation).

Data inclusion is based on spectral quality assessment after baseline correction. Spectra exhibiting excessive residual noise or fluorescence background that obscures diagnostic Raman bands are excluded from further analysis.

All analyses are performed using MATLAB and OriginPro software, and results are visualized using standard plotting and statistical tools. OriginPro is used for baseline correction and spectral range truncation, whereas MATLAB is used for CWT-based peak enhancement, peak detection, and subsequent spectral data processing.

## Limitations

This protocol requires a relatively long sample preparation time, as optimal Raman spectral quality is achieved after extended incubation (approximately 60 days). Therefore, it may not be suitable for time-sensitive applications requiring rapid data acquisition. In addition, because the conditioning solution contains 50% (v/v) glycerol, spectral interpretation should rely primarily on well-established diagnostic Raman bands consistently reported in multiple literature sources.

This protocol does not evaluate the potential Raman contribution of residual glycerol or the molecular mechanisms by which prolonged glycerol conditioning influences spectral quality. Spectral regions with potential overlap between glycerol and plant cell wall vibrations should be interpreted with caution. Furthermore, this protocol provides peak-based semi-quantitative information derived from predefined diagnostic Raman bands and does not determine the absolute concentrations of plant cell wall components; therefore, absolute component concentrations require further validation using complementary analytical approaches.

Protocol performance may vary depending on the physical and biochemical properties of the plant material, including tissue dryness, structural integrity, and inherent spectral and structural differences among samples. Highly degraded or heterogeneous samples may yield reduced spectral quality.

Raman spectral quality can also be influenced by environmental and instrumental factors, such as fluorescence background, laser stability, and minor variations in acquisition settings. Although preprocessing steps can reduce these effects, residual variability may still impact downstream analysis. In addition, some instrument-specific parameters may require optimization and should be reported or adjusted according to the Raman system used.

The protocol was developed using a 532 nm excitation laser. Although Raman peak positions are independent of excitation wavelength, changes in excitation wavelength may influence signal intensity and fluorescence background; therefore, acquisition parameters may require adjustment when using other excitation sources.

## Troubleshooting

### Problem 1

Poor penetration of glycerol solution into dried stem tissues during incubation (Step 5).

### Potential solution

Ensure complete immersion in 50% (v/v) glycerol solution. Gentle agitation during the initial days of incubation can further improve solution infiltration.

### Problem 2

Sample discoloration or microbial contamination during long-term incubation (Step 5).

### Potential solution

Immediately transfer samples to fresh 50% glycerol solution when color changes are observed. Ensure that containers are properly sterilized and tightly sealed before incubation at 4 °C.

### Problem 3

Weak Raman signal or low signal-to-noise ratio during spectral acquisition (Step 6).

### Potential solution

Optimize laser focus on the sample surface and adjust acquisition parameters, including integration time and laser power. If additional flexibility in measurement scheduling is required, samples may remain in the conditioning solution for longer than 60 days without substantial changes in spectral quality, provided that the solution is replaced if discoloration occurs (Figures 4 and 5).

### Problem 4

Strong fluorescence background interfering with Raman peaks (Step 7).

### Potential solution

Apply baseline correction during spectral preprocessing to remove background fluorescence. If desired, CWT-based peak enhancement may be applied to facilitate peak identification by improving the discrimination of closely spaced Raman bands prior to peak detection. If fluorescence persists after baseline correction, adjust the spectral acquisition parameters or restrict the analysis to less affected spectral regions (0–3500 cm^−1^).

Alternatively, the use of longer-wavelength excitation lasers (e.g., 632.8, 785, or 1064 nm) can substantially reduce fluorescence interference by minimizing the excitation of fluorescent compounds in plant tissues. However, the higher cost and limited availability of these laser systems may restrict their routine application.

### Problem 5

Inconsistent Raman spectral marker assignment or spectral variability across samples (Step 8).

### Potential solution

Use standardized reference libraries (Table 2) for Raman spectral marker interpretation and apply consistent peak-picking criteria across all samples (Figure 7).

**Figure 7:**
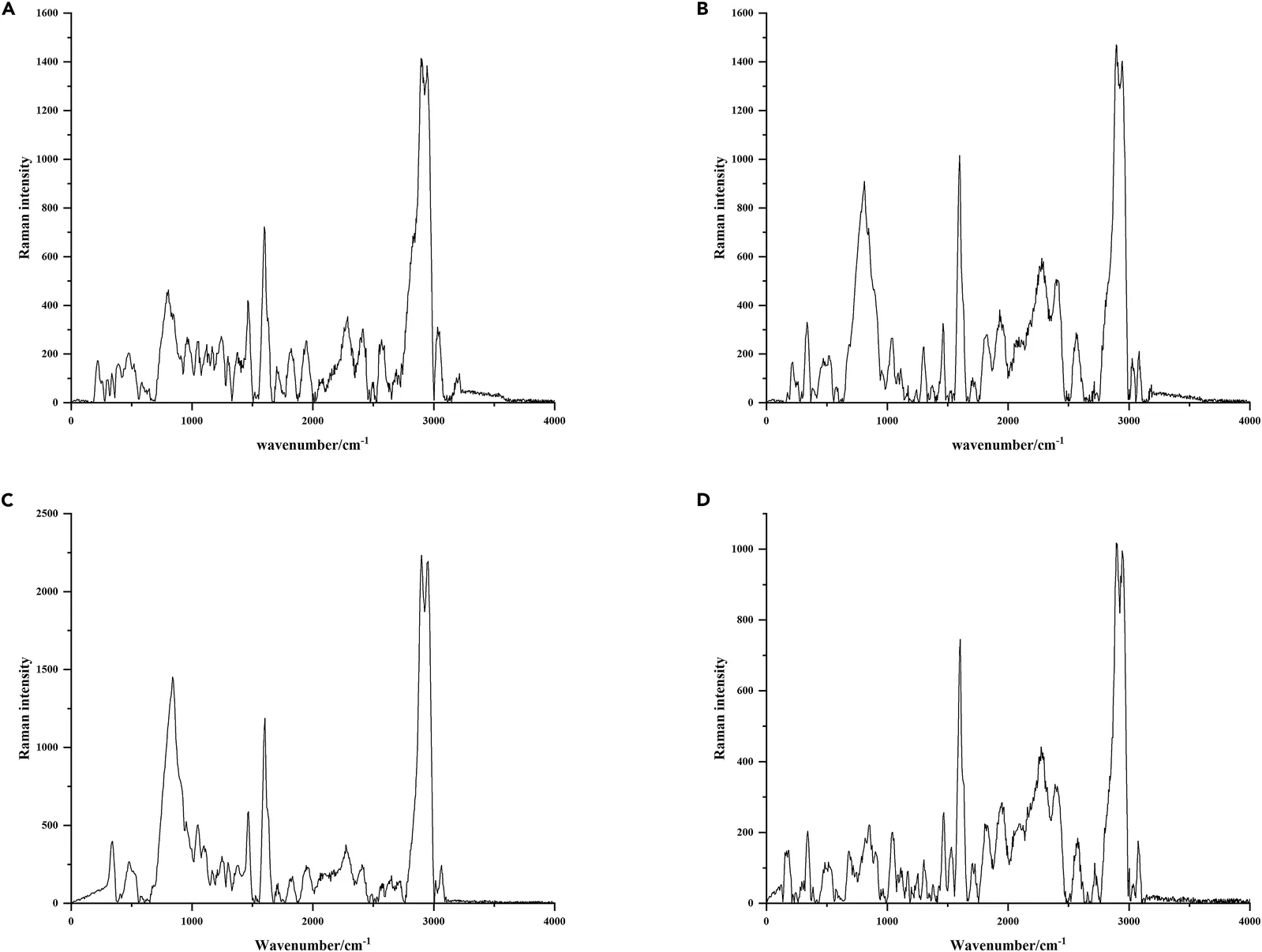
Representative Raman spectra obtained from the cell walls of additional rice straw samples (A) g37, (B) g47, (C) g50, and (D) g70 (see Table S1).

## Resource availability

### Lead contact

Further information and requests for resources and reagents should be directed to and will be fulfilled by the lead contact, Pär K. Ingvarsson (par.ingvarsson@slu.se).

### Technical contact

Technical questions on executing this protocol should be directed to and will be answered by the technical contact, Mahta Mohamadiaza (m_mohamadiaza@sbu.ac.ir).

### Materials availability

This protocol does not generate any unique reagents or materials that require restricted access. All materials used are commercially available and can be obtained from standard suppliers.

### Data and code availability

This protocol describes the data generation and analysis workflows for Raman spectroscopic datasets. The datasets supporting this study are available from the corresponding author upon reasonable request.

Standard software, including MATLAB and OriginPro, were used for data processing and analysis. Custom MATLAB scripts used for spectral preprocessing, including baseline correction and optional CWT-based peak enhancement for peak identification, are provided within this protocol. The CWT implementation is intended for feature enhancement and peak detection rather than wavelet denoising or spectral smoothing.

## Acknowledgments

The authors thank Dr. Mona Shoaei-Parchin (Department of Civil Engineering, Faculty of Engineering, University of Mohaghegh Ardabili) for her assistance in developing the MATLAB code. The Gemological Center of the Faculty of Geology, Shahid Beheshti University, is gratefully acknowledged for providing access to facilities and laboratory space for RS.

## Author contributions

M.M. carried out sample harvesting; performed sample preparation; designed and executed the experiments; conducted data quantification, validation, and analysis; and drafted the original manuscript. N.F. conceptualized the study, planned the experiments, supervised the research, and reviewed and edited the manuscript. P.K.I. advised on methodology and data analysis, provided resources, and reviewed and edited the manuscript. A.A. co-supervised the study, developed the code required for data analysis, and reviewed and edited the manuscript.

## Declaration of interests

The authors declare no competing interests.

## Supplemental information

Document S1. Figure S1

Table S1. Information on rice genotypes studied during the validation step, related to Troubleshooting, Problem 5

Table S2. Genotype information for rice samples, related to Figure 1

Table S3. Baseline-corrected Raman spectral data used for downstream analysis, related to Step 8

Data S1. MATLAB code for Raman peak enhancement and detection

Data S2. Data input for MATLAB code
