# Supplementary material for: Rice straw sample preparation protocol for cell wall major constituent analysis via Raman spectroscopy
Source: STAR Protoc. 2026 Sep 26;7(4):104857. doi: 10.1016/j.xpro.2026.104857 (PMC13639168; doi:10.1016/j.xpro.2026.104857)
Supplement: Supplementary file 1 [file mmc1.pdf]

Figure S1. Experimental workflow

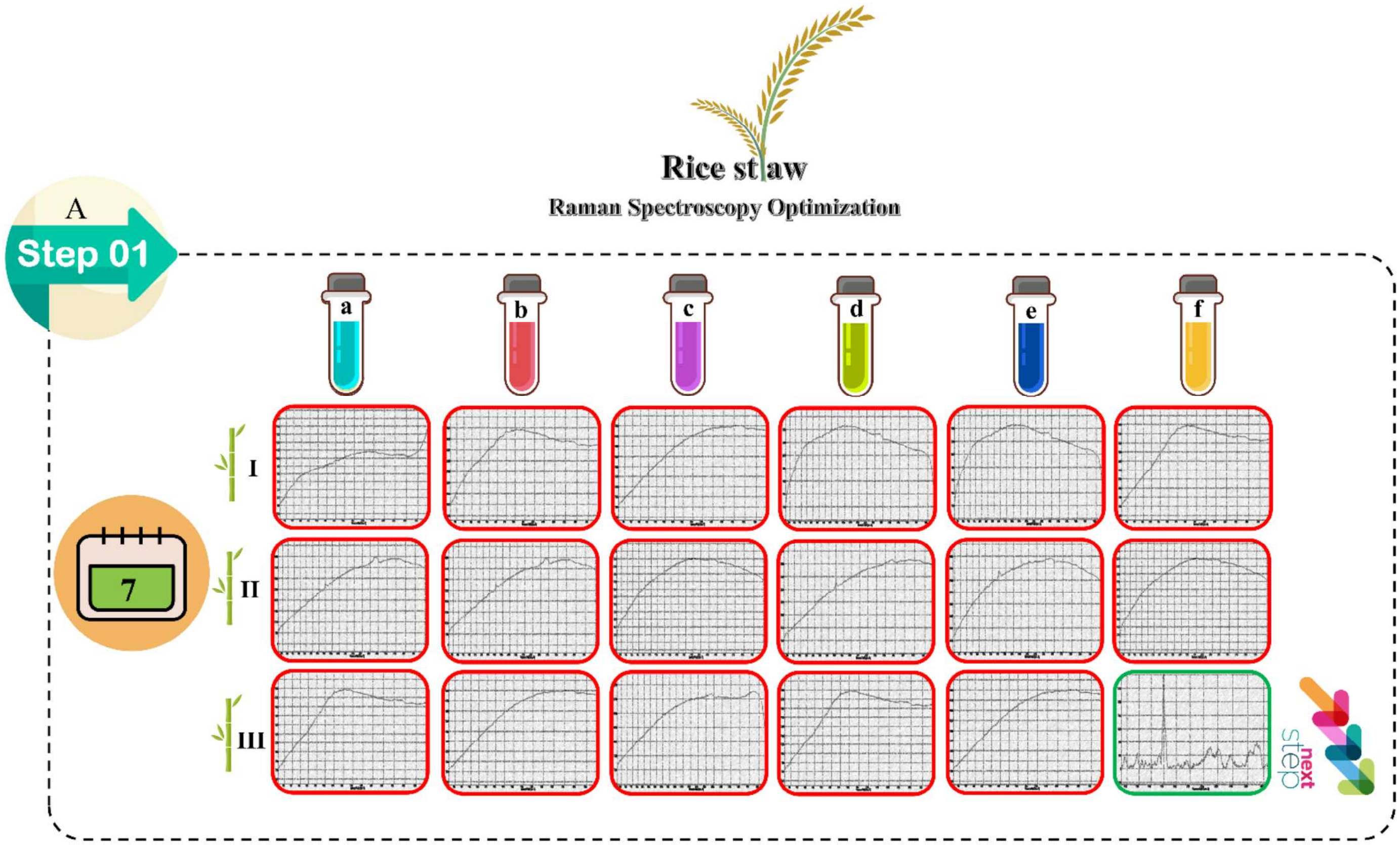

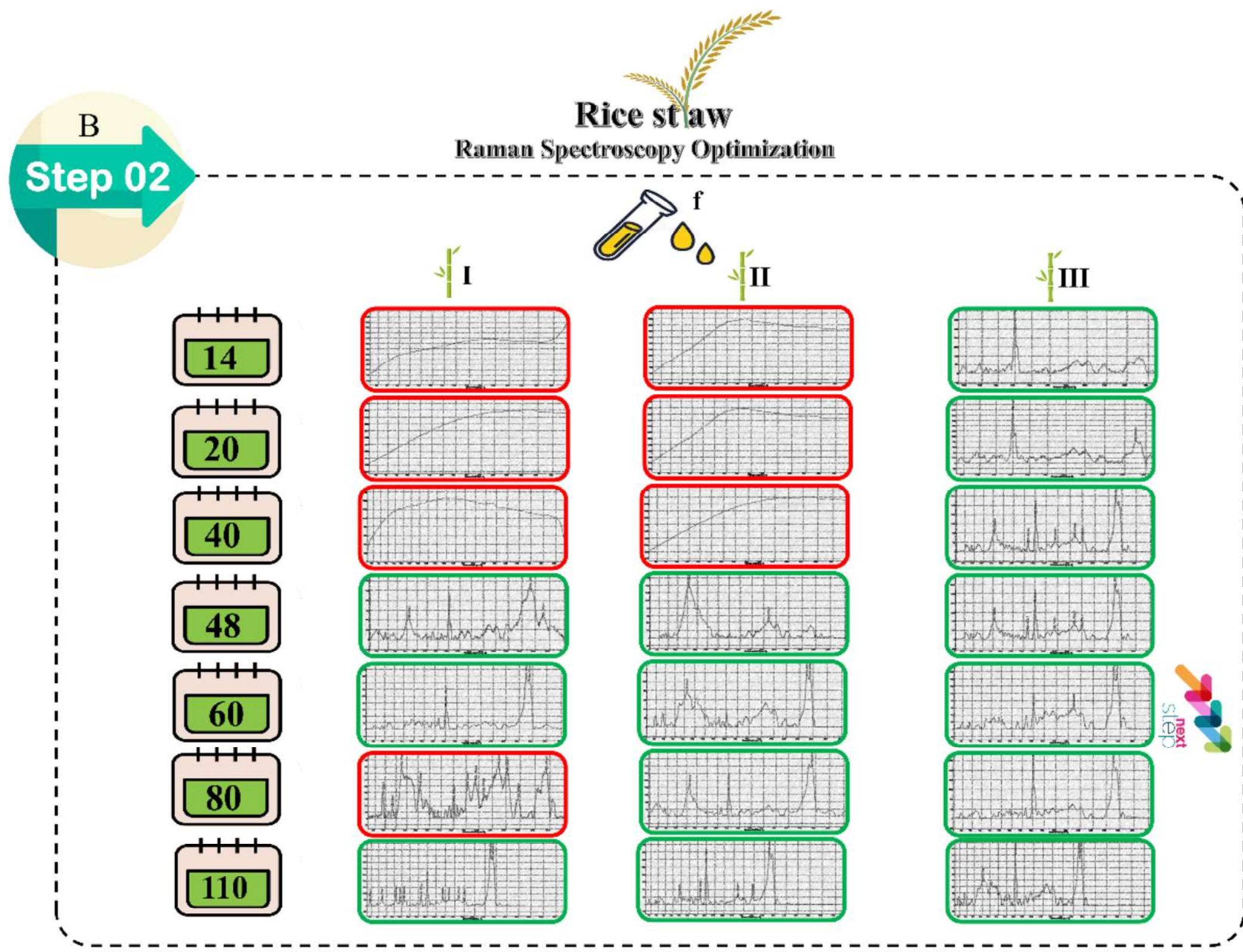
B
Step 02
Rice st aw
Raman Spectroscopy Optimization
f
I
II
III
14
20
40
48
60
80
110

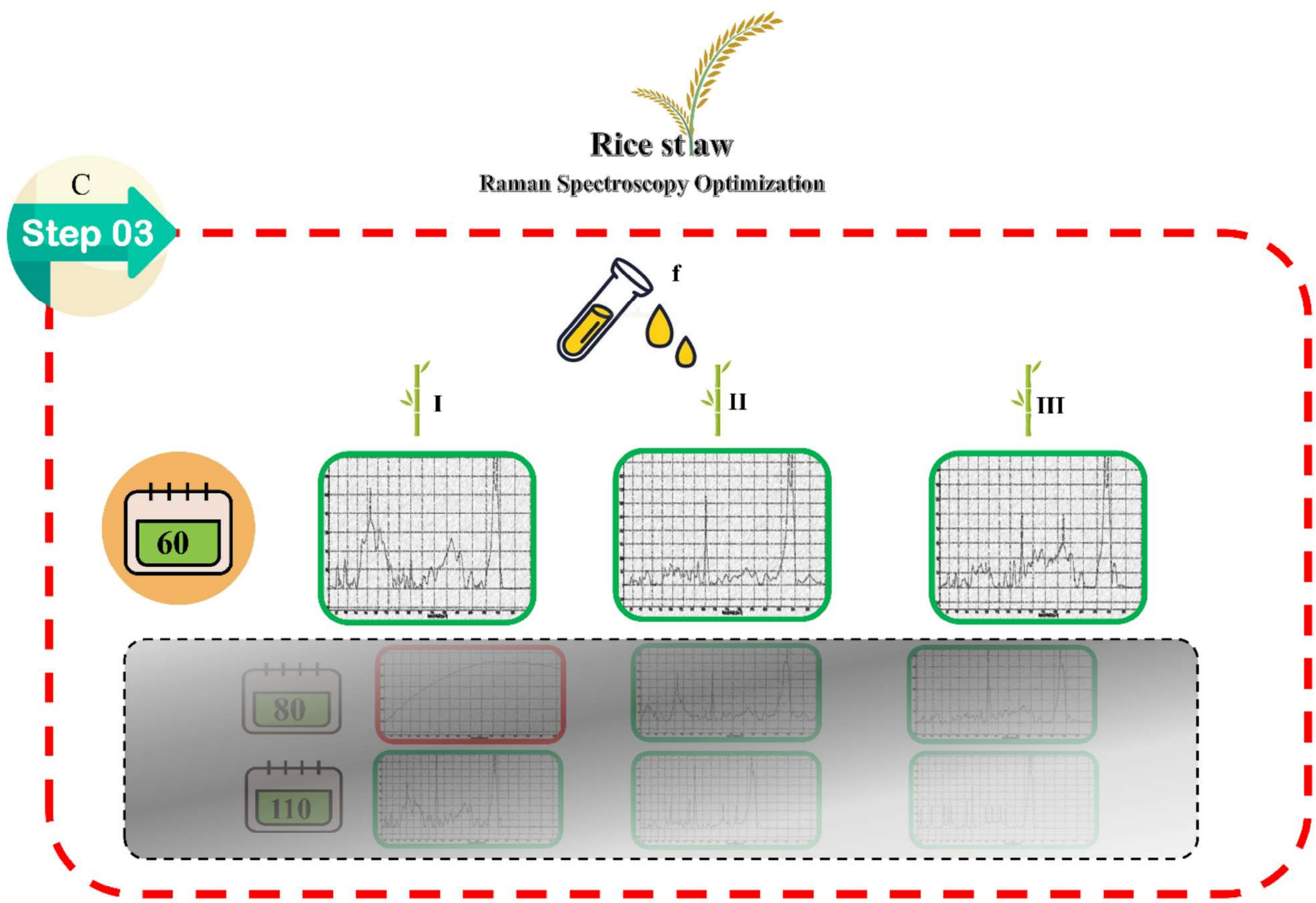
C
Step 03
Rice st aw
Raman Spectroscopy Optimization
f
I
II
III
60
80
110

Figure S1. Experimental workflow for optimizing the conditioning solution prior to the development of the Raman sample preparation protocol. (A) Three rice stem samples (g236, g57, and g135), collected from the third internode, were incubated for 7 days at 4 °C in six different conditioning solutions: (a) distilled water, (b) 70% ethanol, (c) fixative solution (50% ethanol, acetic acid, and 37% formalin), (d) toluene following paraffin embedding and deparaffinization, (e) pure glycerol, and (f) 50% (v/v) glycerol. Raman spectra obtained from all treatments were compared, and only the samples conditioned with 50% glycerol yielded detectable Raman spectral features suitable for further optimization. Consequently, 50% (v/v) glycerol was selected for the subsequent experiments, in which the effect of incubation time on Raman spectral quality was investigated (Figure 5). (B) Optimization of the incubation period. Following the selection of 50% (v/v) glycerol as the conditioning solution, the solution composition and sample type were kept constant while the incubation time was varied (14, 20, 40, 60, 80, and 110 days; see also Figure 5). This experiment was designed to evaluate the effect of incubation duration on Raman spectral quality, including the clarity and reproducibility of characteristic Raman peaks, and to determine whether prolonged conditioning enabled the detection of additional cell wall-related spectral features. (C) Selection of the optimal conditioning period. Comparison of Raman spectra obtained after different incubation periods demonstrated that spectral quality progressively improved up to 60 days, with no substantial additional improvement observed after 80 or 110 days. Therefore, incubation in 50% (v/v) glycerol at 4 °C for 60 days was selected as the optimal conditioning protocol, as it represented the shortest incubation period that consistently produced stable, high-quality Raman spectra while avoiding unnecessary prolongation of sample preparation. The inclusion of the 80- and 110-day time points further confirmed that samples remained spectrally stable during extended storage under the same conditioning conditions.
